# Secretion of Human Protein C in Mouse Milk

**DOI:** 10.3390/ijms16034904

**Published:** 2015-03-04

**Authors:** Chae-Won Park, Myung-Hwa Kang, Kwan-Sik Min

**Affiliations:** 1 Animal Biotechnology, Graduate School of Bio and Information Technology, Institute of Genetic Engineering, Hankyong National University, Ansung 456-749, Korea; E-Mail: mol517@daum.net; 2 Department of Food and Nutrition, Hoseo University, Asan 336-795, Korea; E-Mail: mhkang@hoseo.ac.kr

**Keywords:** human protein C, transgenic mice, mammary glands

## Abstract

To determine the production of recombinant human protein C (rec-hPC) in milk, we created two homozygous mice lines for the goat β-casein/hPC transgene. Females and males of both lines (#10 and #11) displayed normal growth, fertility, and lactated normally. The copy number of the transgene was about fivefold higher in #10 line as compared to #11 line. mRNA expression of the transgene was only detected in the mammary glands of both lines. Furthermore, mRNA expression was fourfold higher on day 7 than on day 1 during lactation. Northern blot analysis of mRNA expression in the #10 line of transgenic (Tg) mice indicated a strong expression of the transgene in the mammary glands after seven days of lactation. Comparison of rec-hPC protein level with that of mRNA in the mammary glands showed a very similar pattern. A 52-kDa band corresponding to the hPC protein was strongly detected in mammary glands of the #10 line during lactation. We also detected two bands of heavy chain and one weak band of light chain in the milk of the #10 and #11 lines. One single band at 52 kDa was detected from CHO cells transfected with hPC cDNA. hPC was mainly localized in the alveolar epithelial cell of the mammary glands. The protein is strongly expressed in the cytoplasm of the cultured mammary gland tissue. hPC protein produced in milk ranged from 2 to 28 ng/mL. These experiments indicated that rec-hPC can be produced at high levels in mice mammary glands.

## 1. Introduction

Protein C, also known as autoprothrombin IIα and blood coagulation factor XIV, is a zymogenic (inactive) protein, which in its active form plays an important role in regulating blood clotting, inflammation, cell death, and maintaining blood vessel permeability in humans and other animals [1]. Its structure is that of a two-chain polypeptide consisting of a light chain and a heavy chain connected by a disulfide bond. This protein is a vitamin K-dependent plasma protein that is synthesized in the liver as a 461 amino acid serine protease precursor and undergoes several co- and/or post-translational modifications, including endoprotease cleavage into disulfide-linked heavy (Mr 41 kDa) and light (Mr 21 kDa) chains [2]. Activated protein C (APC) performs these operations primarily by proteolytically inactivating proteins Factor Vα and Factor VIIIα [3]. Given the crucial role that protein C plays as an anticoagulant, those with deficiencies in protein C, or a resistance to APC, suffer from a significantly increased risk of forming dangerous blood clots (thrombosis). Transgenic animals that produce recombinant proteins in their milk can provide an economical and safe system for the production of medicinally valuable proteins [4,5]. Many attempts have been made to find an alternative production system for hPC, and several groups have explored the possibility of expressing hPC in the milk of transgenic mammals [6,7,8,9,10,11]. However, thus far, hPC Tg mice failed to lactate normally and were unable to raise litters [12].

Several medical proteins have been successfully produced using the goat promoter system. As one of the dominant milk proteins, β-casein is thus implicated in determining milk levels. Using the goat β-casein promoter, therapeutic proteins have been expressed at high levels in the milk of Tg mice [13]. Protein C is likely to have multiple biomedical functions as a result of anticoagulant activity. In addition to treating protein C deficiency, it has been shown to be useful in the treatment of coumarin-induced skin necrosis [14], coagulopathology in meningococcal disease [15], immediate and transient inhibition of platelet-dependent arterial thrombosis [16], treatment of stroke, and in the prevention of reocclusion in patients treated with fibrinolytic agents [17]. For these reasons, efforts are being made to isolate large amounts of protein C. In this study, we focused on the production of hPC in the milk of hPC Tg mice. We demonstrated that hPC is localized in the alveolar epithelial cells within the mouse mammary gland, and that these glands secrete hPC into the milk.

## 2. Results

### 2.1. Production of hPC Transgenic Mice

**Figure 1:**
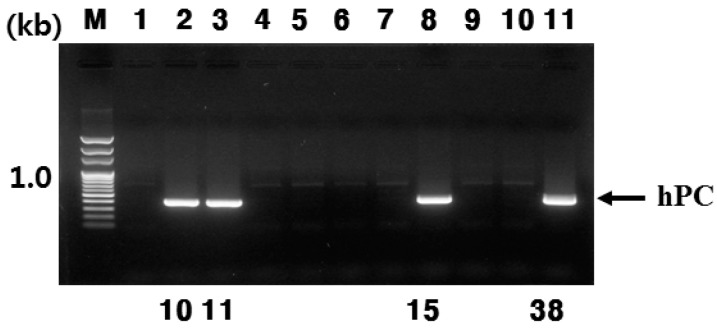
Detection of human protein C (hPC) transgene in founder mice by PCR analysis. Four transgenic mice were identified by transgene specific primers. PCR products are placed on the right. Sizes of DNA markers are on the left (in nucleotides). Lane 1: Negative control with DNA; Lane 2–11: Samples extracted from tails. Number 10, 11, 15, and 38 was detected by PCR as expected size. M: Marker.

In total, 469 one-cell embryos were transferred to 24 recipient mice. Sixty-one pups were born from 22 recipients that had received embryos microinjected with the pBC1-hPC construct. Screening of genomic DNA from tail biopsies using PCR indicated that four litters contained the transgene, for an integration frequency of 6.6%. Four transgenic mice were identified by transgene-specific primers for genomic DNA, which was expected to produce 470 bp products (Figure 1).

**Figure 2:**
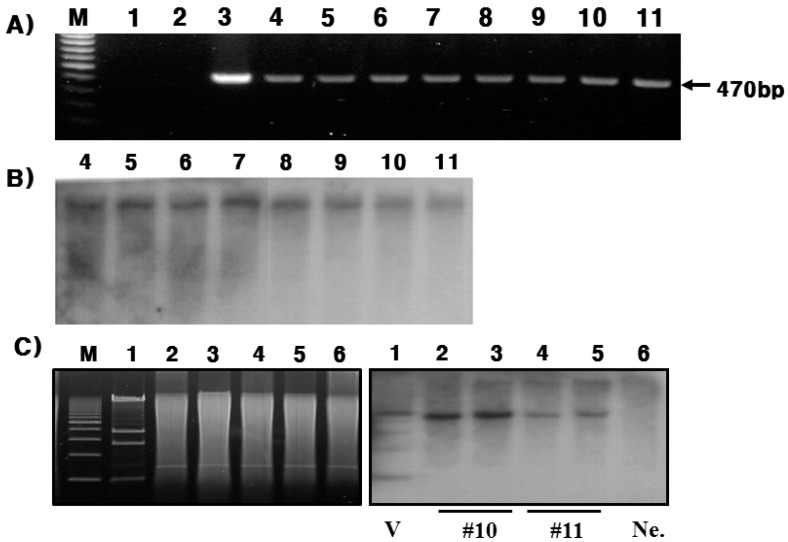
Transmission into the germline from transgenic mice. DNA was isolated from tail biopsies. DNA samples were digested with KpnI, run on 0.7% agarose gel, transferred to nitrocellulose membranes, and hybridized with hPC cDNA. (**A**) The second to fifth generations of transgenic mice were amplified by PCR from #10 and #11 lines. Lanes 1: Negative control without DNA; Lanes 2: Negative control with DNA; Lane 3: Positive control with hPC cDNA vector; Lanes 4–7: #10 line; Lanes 8–11: #11 line; (**B**) Southern blot results from heterozygotes DNA samples. Number 4–7: #10 lines; Number 8–11: #11 lines; (**C**) Southern blot results in the fifth generation of homozygous Tg mice were analyzed from #10 and #11 lines. Electrophoresis results from KpnI enzyme cut (left). Number 2–3: #10 line; Number 4–5: #11 line. V: Constructed vector; Ne.: Negative Control. M: Marker.

The transgenic lines for four positive founders (#10, #11, #15, and #38) were produced. The screen was confirmed by PCR and Southern blotting analysis (Figure 2A,B). Southern blot analysis of tail DNA from line 10 and 11 is shown in Figure 2C. The hPC gene was inserted into the chromosomes in the #10 line and the #11 line. The copy number of the transgene was approximately fivefold higher in the #10 line as compared with the #11 line (Figure 2C). Homozygous Tg mice were produced to the F5 generation. Thus, we confirmed that these two lines (#10 and #11) were transmitted into the germline.

### 2.2. Evaluation of mRNA Expression by RT-PCR, qRT-PCR, and Northern Blot

**Figure 3:**
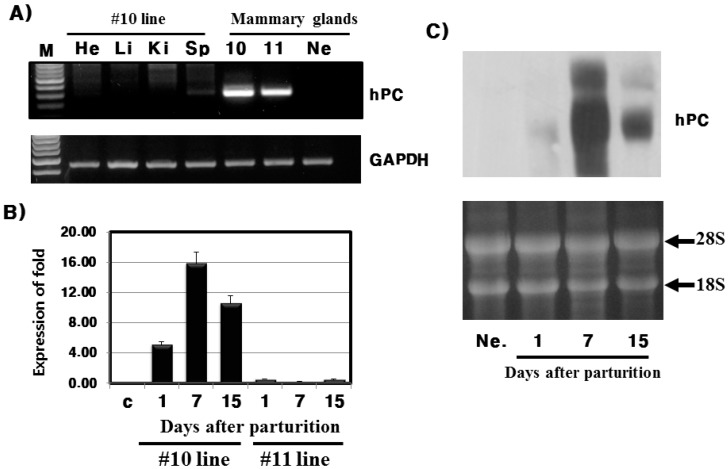
mRNA expression by RT-PCR, qRT-PCR, and Northern blot. (**A**) RT-PCR results from mammary glands and other tissues; (**B**) qRT-PCR results from mammary glands on days 1, 7, and 15 during lactation; (**C**) Northern blot probed with hPC cDNA in the #10 line. M: marker; He: heart; Li: liver; Ki: kidney; Sp: spleen; Ne: negative control.

Using the specific primers for hPC, RT-PCR and qRT-PCR was conducted to detect mRNA expression in mice tissues. hPC was only detected in the mammary glands of Tg mice from #10 and #11 lines (Figure 3A). There was a small amount of hPC expression in the spleen of the #10 line, which may be due to in PCR. However, we did not detect any expression of hPC in the heart, liver, and kidney. The qRT-PCR analysis was also performed for hPC in the mammary glands on days 1, 7, and 15 during lactation. In the #10 line, the expression level of hPC was fourfold higher on day 7 compared to day 1. However, this protein was rarely expressed in the #11 line, as evidenced by the qRT-PCR results (Figure 3B). Thus, the inserted hPC gene in the #11 line may be unstable, mosaic, and not produce rec-hPC. Northern blot analysis of mammary gland RNA for the #10 line Tg mice and control mice were conducted with Dig-labeled hPC. A very strong band, about 1 kb size, was detected seven days after parturition (Figure 3C).

### 2.3. Western Blot Analysis in the Mammary Glands and Milk

**Figure 4:**
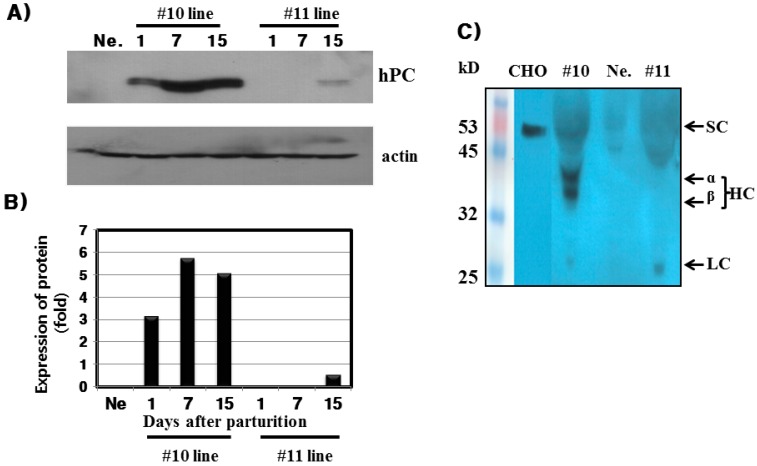
Western blot analysis of hPC from the mammary glands and milk during lactation. (**A**) Protein detection from mammary glands during lactation; (**B**) The quantity analysis of hPC expressed in the mammary glands; (**C**) hPC protein detection assessed in the milk of the #10 and #11 lines via Western blot. SC: single-chain hPC forms; HC: heavy-chain hPC form; LC: light-chain hPC forms; CHO: hPC expressed into the CHO cells transfected with hPC cDNA.

As shown in Figure 4A, a 52-kDa band corresponding to hPC protein was strongly detected in the mammary glands of the #10 line Tg mice on days 1, 7, and 15 during lactation. A weak signal was detected in the #11 line Tg mice on day 15, but no signal was detected on days 1 and 7. hPC expression was remarkably high on days 7 and 15 during lactation. When compared, the pattern in mRNA expression was very similar to the pattern of protein expression in the mammary glands. Furthermore, rec-hPC protein was also assessed in the milk of the #10 and #11 lines via Western blot. We detected two bands of heavy chain and one weak band of light chain in these two Tg mice lines. One single band of 52-kDa was also detected in the CHO cell transfected by hPC cDNA (Figure 4C).

### 2.4. Localization of hPC Protein in the Mammary Gland

**Figure 5:**
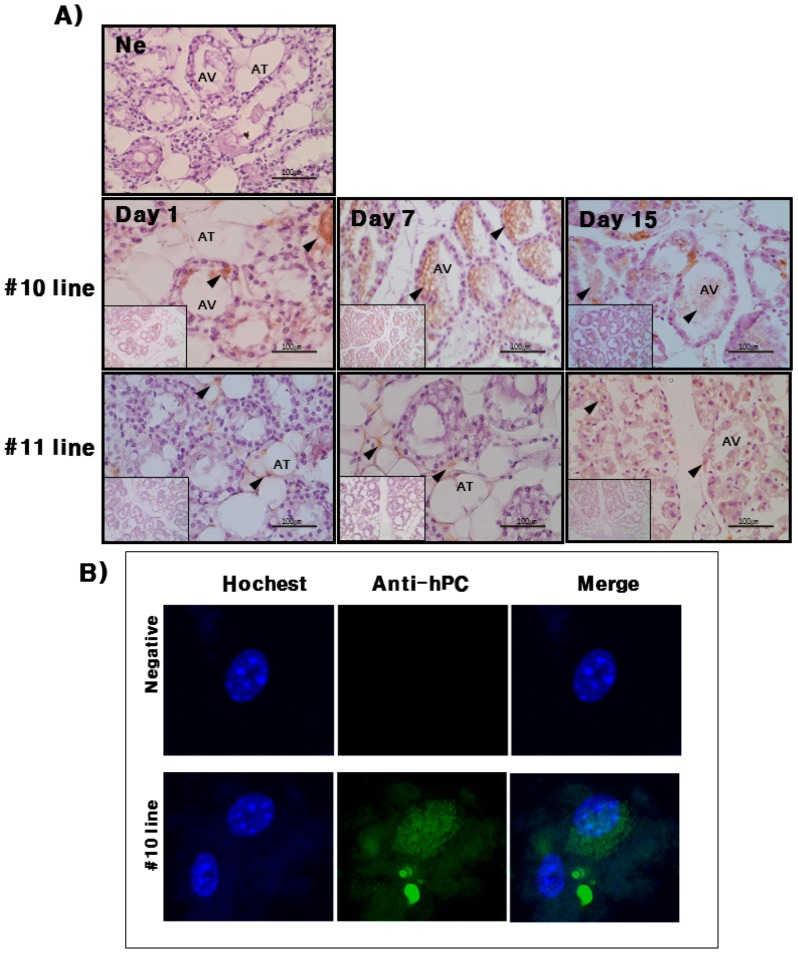
Localization of hPC protein expressed in the mammary glands during lactation by immunohistochemistry and in the cultured Tg mice mammary gland cells by immunofluorescence. (**A**) Immunohistochemistry. Representative immunohistochemical analyses using hPC antiserum (1:1000) and swine anti-rabbit secondary antibodies (1:1000). Preimmune serum (1:1000) was used for primary antiserum as the negative control. Mammary gland sections are shown on days 1, 7, and 15 during lactation. Black bar = 100 μm. Arrows indicate alveolar epithelial cells of the mammary gland; (**B**) Immunofluorescence in cultured mammary glands of Tg mice (#10 line) on day 7 during lactation. Counter staining was performed with DAPI (blue). hPC expression was detected by Alexa 488 (green). The merged picture shows blue and green colors.

To determine the cell types that are responsible for hPC protein expression in the mammary gland, we performed immunohistochemical analysis on days 1, 7, and 15 during lactation in #10 and #11 line Tg mice. As shown in Figure 5A, Tg mice expressing hPC protein were mainly localized in the alveolar epithelial cells of the mammary gland. We also determined the localization of hPC in cultured cells from the mammary gland tissues on day 7 during lactation. The data shown in Figure 5B indicate that this protein is strongly expressed in the cytoplasm of cultured cells that originated from Tg mice mammary glands.

### 2.5. Enzyme-Linked Immunosorbent Assay (ELISA)

**Figure 6:**
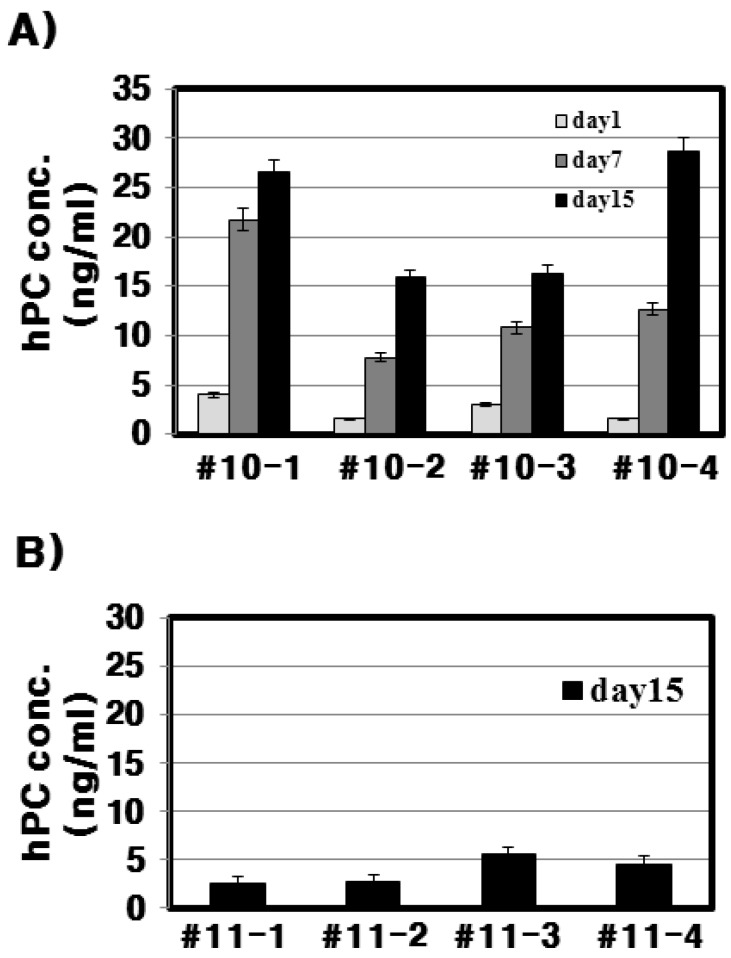
The quantity of rec-hPC in the Tg mice milk by ELISA. (**A**) rec-hPC in the #10 lines analyzed by ELISA on days 1, 7, and 15 during lactation; (**B**) hPC analysis in the #11 line. Each point and vertical bar represents the mean and standard error of the mean (SE) values, respectively, for the three different samples.

To analyze the quantity of rec-hPC protein in the Tg mice milk, we performed an ELISA on days 1, 7, and 15 during lactation. As shown in Figure 6, rec-hPC in the #10 line Tg mice ranged from 2 to 28 ng/mL It was under 4 ng/mL on day 1 and then it was greatly increased on day 7, and ranged from 17 to 28 ng/mL on day 15. Conversely, analysis of the milk from the #11 line on day 15 indicated that all samples were less than 5 ng/mL.

## 3. Discussion

In the present study, we successfully produced hPC in the mammary glands and milk of Tg mice. This was confirmed through RT-PCR, Northern blotting, and immunohistochemistry during lactation. In this study, the goat β-casein promoter system was used for expressing the hPC gene. We established four positive transgenic mice, denoted #10, #11, #15, and #38. However, only #10 and #11 lines of Tg mice were able to bred (Figure 6A,B). Females of both lines (#10 and #11 lines) had normal growth and fertility. However, the #15 and #38 lines were unable to breed. This may be the reason for the mosaicism of the inserted gene. Previous studies have observed something similar with Tg mice expressing the hPC gene failing to lactate normally or raise litters [12]. These defects were not found in the fifth to eighth generation Tg mice produced in this study (data now shown). Our integration frequency in the founder mice was 6.6%, and that was with only one injection. In total, 26 pups were obtained from eight recipients for an integration frequency of 27% [11]. However, a similar integration frequency as seen in this study was observed in Tg mice expressing dimeric human erythropoietin [18].

Analysis of mRNA expression indicates that expression was specific to the mammary glands and was the highest on day 7 during lactation. These results are very similar to previous studies that reported a spike in hPC mRNA expression in the mid-lactation [8], and on day 10 of lactation [6] for hPC Tg mice. In pig, hPC mRNA was also highly expressed during early lactation and on day 35 and 55 [11]. Furthermore, mRNA expression of human growth hormone under the bovine β-casein gene promoter was only present in the mammary glands of lactating bovine [19]. Moreover, Ebert *et al.* [20] suggest that mammary glands contain highly expressed levels tissue plasminogen activator to the termination of 30–89’s third lactation in goats. Thus, we concur that the use of the β-casein promoter may be useful for material production in milk.

hPC protein expression was strongly detected in the mammary glands of the #10 Tg mice. Its expression was the highest on day 7 during lactation. For the rec-hPC protein expressed in the milk, both the heavy chain and light chain was observed at approximately 41 and 21 kDa, respectively. In addition to these two bands, there was a single band at 52 kDa. These observations were consistent with previous reports for rhPC expression [6,8,9,11,12]. The molecular mass of the putative heavy chain forms of rec-hPC (35–38 kDa), light chain (18–20 kDa), and single chain of rec-hPC (about 55–58 kDa) were about 2–3 kDa lower than that of hPC [11]. This may be due to differences in carbohydrate content and structure. The rec-hPC contained a significantly larger population of single chain material than naturally expressed hPC, which may indicate a rate limitation in the post-translational removal of the dipeptide Lys-Arg at positions 156 to 157 [21]. rhPC protein was intensively localized to alveolar epithelial cells of the mammary gland. We also determined through immunofluorescence that expression of rec-hPC was localized in the cytoplasm of cells cultured from mammary glands on day 7 of lactation. This observation is consistent with previous data showing the presence of rec-hPC protein in the secretory epithelium, and alveolar and ductal lumina [8,12]. Thus, we suggest that rec-hPC is correctly targeted and undergoes normal secretion by mammary epithelial cells. Our results demonstrate that expression of the hPC gene does not have obviously any obvious toxic effect on the mammary glands of Tg mice.

With respect to the #10 line Tg mice, the amount of rec-hPC secreted into the milk on day 15 of lactation, as determined by ELISA, was 15–28 ng/mL and on day 7 (8–22 ng/mL). However, the amount of rhPC in the # line 11 was under 5 ng/mL. The hPC amount in F_o_ females, as determined by ELISA, was 1–75 μg/mL, which was lower than in later generations (0.31–1.53 mg/mL). This may be due to mosaicism of the founders [6]. The genomic hPC in mice was increased about 200-fold from 0.5–3 μg/mL in the mice with the cDNA construct [11] to 0.1–0.7 mg/mL in the mice with the genomic construct [22]. In pigs, both cDNA- and genome-derived hPC was secreted in similar amounts (100–1800 μg/mL), and the expression level ranged between 100–400 μg/mL in the outbred lineage for the cDNA transgene [20]. Tg pigs were generated that produced rec-hPC in their milk at up to 1 g/L [11]. The levels of rec-hPC in the milk ranged from 40–1200 μg/mL [9]. These expression differences in Tg lines may occur due to differences in promoter used, gene construction, mosaicism of inserted gene, and gene type. In mice, expression of hPC induced an abnormal lactation phenotype [4,12] and a slight phenotype was reported [23]. In the present study, we did not find any phenotype changes up to the eighth generation.

## 4. Experimental Section

### 4.1. Transgenic Mouse Vector Construction for hPC

hPC cDNA was amplified by polymerase chain reaction (PCR) with human liver cDNA using the forward primer for hPC (5'-CA GGT ACC ATG TGG CAG CTC ACA AGC CTC CTG-3'), which contains the SalI site at the 5' end, and the reverse primer for hPC (5'-TAC TCG AGC CTA AGG TGC CCA GCT CTT CTG GGG-3'), which contains the XhoI site at the 3' end (Takara, Osaka, Japan). The amplified products were cloned into pCR2.1 vector and then completely sequenced to check for errors. The resulting fragments were digested by SalI/XhoI and ligated into the unique XhoI site of the transgenic expression vector under the control of the goat β-casein promoter (designed as pBC1-hPC) as previously reported [18]. The direction of the ligated fragment was confirmed by restriction mapping using XhoI and SalI enzymes. The sequence of the entire hPC cDNA was verified by automated DNA sequencing performed as previously reported [18].

### 4.2. Production and Screening of Transgenic Mice

Tg mice were obtained by pronuclear microinjection of the hPC cDNA driven by the goat β-casein promoter, as previously described [24]. Briefly, NotI/SalI fragments were purified by the phenol extraction method and injected into one-celled fertilized embryos. C57BL/6N mice were used for the experiment. All mice were raised and maintained in the facilities of Hankyong National University. Genomic DNA was extracted from offspring tail biopsies. The PCR primers were as follows: hPC-1 forward primer, 5'-CCC TGA CCA GGG ATC AAA CCT GC-3' and hPC-1 reverse primer, 5'-CCT TGG CCT CCT CGA AGT CAC A-3'. PCR was performed over 35 cycles (1 min at 94 °C, 1 min at 58 °C and 1 min at 72 °C). The predicted PCR product was 470 bp in length. The Tg mice were bred by mating heterozygous male/female mice with wild-type female/males. The homozygous Tg mice were produced by hetero-hetero mating. The experiments were conducted according to the Guidelines for the Care and Use of Laboratory Animals, Hankyong National University (Num: 2014-4).

### 4.3. Southern Blot Analysis

The DNA extracted from the tail of Tg mice was electrophoresed on a 0.7% TBE agarose gel. DNA was then transferred to a nylon membrane. Blots were prehybridized at 60 °C for 1 h and hybridized at 60 °C with overnight rocking (final probe concentration was 20 ng/mL). The membrane was subsequently washed three times in 1× SSC/0.1% SDS at 68 °C for 5 min each time, and then three times in 0.1× SSC/0.1% SDS at 68 °C for 5 min each time. An anti-Dig antibody tube was centrifuged at 13,000 rpm for 5 min at 4 °C to remove the pellet. A 1.5 μL aliquot of anti-Dig antibody was then added to the blocking reagent and membrane bag, and incubated at RT for 1 h. Following incubation, the membrane was washed three times in TBST at RT for 10 min each time. Finally, DNA bound to the membrane was detected with CDP-star.

### 4.4. Reverse Transcriptase-Polymerase Chain Reaction (RT-PCR) Analysis

Isolation of total RNA from frozen Tg mice heart, liver, kidney, spleen, and mammary gland tissues was performed using the TRIzol reagent (Invitrogen, Carlsbad, CA, USA) according to the manufacturer’s specifications. The final RNA sample was treated with DNase to prevent DNA contamination. For RT-PCR analysis, the reverse transcription reaction was performed using 1 μg of total RNA using SuperScript II Reverse Transcriptase and primers (Invitrogen, USA) according to the manufacturer’s protocols. The cDNA synthesis reaction was performed using the following protocol: 42 °C for 60 min and 94 °C for 5 min. PCR was carried out according to the following protocol: 94 °C for 1 min, followed by 30 cycles of 94 °C for 1 min, 55 °C for 1 min, and 72 °C for 1 min). The hPC gene was detected using a forward primer (5'-GCA GCG AGG TCA TGA GCA AC-3') and reverse primer (5'-AAG AAT AGG GAA GGG TCC-3'), which yielded 292 bp DNA fragments. Glyceraldehyde 3-phosphate dehydrogenase (GAPDH) was used for normalization: forward primer was 5'-ACC ACA GTC CAT GCC ATC AC-3' and reverse primer was 5'-TCC ACC ACC CTG TTG CTG TA-3'. The expected PCR fragment should have a length of 452 bp. The PCR conditions for GAPDH were 26 cycles of 10 s at 98 °C, 30 s at 55 °C, and 30 s at 72 °C.

### 4.5. Quantitative Real-Time PCR (qRT-PCR)

qRT-PCR was described previously [18]. Two micrograms of total RNA was reverse transcribed to cDNA using Oligo(dT) primers with the first-strand cDNA synthesis kit for RT-PCR (Invitrogen) according to the manufacturer’s instructions. qRT-PCR was performed in a Rotor-Gene™ 6000 (Corbett Life Science, Mortlake, Australia). The reaction mixture consisted of 100 ng DNA, 4 nM of each primer, 10× qPCR Mastermix Plus (Toyobo, Osaka, Japan) in a total volume of 20 μL. A negative control containing all reagents minus DNA was included in each run. All reactions were performed with an initial denaturation step for 10 min at 95 °C followed by 40 cycles of 95 °C for 10 s, an annealing temperature of 55 °C for 1 min with the same primer used in RT-PCR. The housekeeping gene β-actin was run in parallel (forward primer: 5'-AGA GGG AAA TCG TGC GTG AC-3' and reverse primer: 5'-CAA TAG TGA TGA CCT GGC CGT-3'). The threshold cycle number and reaction efficiency were determined using the Rotor-Gene 6000 series software version 6.1.93; the 2^−∆∆*C*t^ method was used for relative quantitation. Absence of primers and the presence of the correct amplicon size for the specific SYBR green assays were verified by melting-curve analysis.

### 4.6. Northern Blot Analysis

RNA blot analysis was carried out using the total RNA from mammary gland tissues prepared on days 1, 7, and 15 after parturition (Control, 1, 7, and 15). Briefly, RNA electrophoresis was performed on an agarose gel containing 10× 3-(4-morpholino) propane sulfornic acid and 37% formaldehyde, and the total RNA concentration was diluted to 10 μg/μL. After electrophoresis, the RNA was transferred onto a membrane with a 20× saline-sodium citrate buffer and incubated overnight at RT. The probe was prepared by purifying the sample after PCR amplification. Probe labeling was performed by the DIG DNA labeling kit. The membrane was pre-hybridized for 1 h at 68 °C and hybridized at 68 °C overnight with DIG-labeled human protein C cDNA with gentle rocking. The bands were visualized the following day using an antibody conjugate and X-ray film.

### 4.7. Milk Collection and Preparation

Mice litters were removed from their mother for 1 h prior to milking to allow for milk accumulation. Milk letdown was induced by intramuscular administration of five units of oxytocin. Milk was collected on days 1, 7, and 15 of lactation, diluted with two volumes of PBS, and centrifuged at 4 °C for 30 min at 14,000 rpm to separate the whey, casein, and fat fractions. Control mice milk was treated in the same fashion. All samples were stored at −20 °C until used.

### 4.8. Western Blot Analysis

Total proteins were extracted by PRO-PREP™ protein extraction solution. Approximately 10–20 mg of tissue was used. After digging the tissues, the sample was transferred to an appropriate tube and homogenized in 600 μL PRO-PREP™ solution. Finally, the samples were centrifuged at 105,000× *g* at 4 °C for 5 min, and the supernatants were transferred to fresh 1.5 mL tubes. Then protein concentration was measured by Bradford method [25]. After blotting, the membrane was blocked with 1% blocking reagent buffer for 1 h and incubated with a 1:1500 dilution of a primary antibody for 1 h. The membrane was then washed to remove unbound antibody and incubated with a 1:2000 dilution of an HRP conjugated antibody anti-rabbit IgG secondary antibody for 2 h. The membrane was then incubated for 1 min with 2 mL Lumi-Light substrate solution, after which the solution was discarded. The membrane was exposed on X-ray film for 1–10 min.

### 4.9. Immunohistochemistry of Mammary Gland Samples

Immunohistochemistry staining of mammary gland samples were performed by the Vectastain ABC kit (Vector Laboratories, Burlingame, CA, USA). The samples were fixed in 70% DEPC-treated water ethanol at 4 °C for 24 h. The fixed samples were then rehydrated using grade ethanol (10 min each in 100% 2×; 95% 2×, 70% 2×) and embedded in paraffin. Paraffin-embedded tissues were sectioned into 5-μm slices on a microtome, which were then mounted onto poly l-lysine-coated slides. The slides were boiled in 10 mM sodium citrate for 10 min and chilled on ice for 20 min. The slides were then washed in 3% hydrogen peroxide for 10 min, and 5% horse serum was used as the blocking agent for 1 h at room temperature. After washing, the slides were incubated overnight at 4 °C with the primary antibody diluted in 5% horse serum blocking buffer. The slides were then incubated with biotinylated secondary antibody (polyclonal swine anti-rabbit IgG, 1:1000 dilutions). The slides were immunostained using the ABC detection kit and stained with diaminobenzidine (DAB).

### 4.10. Mammary Gland Cell Culture and Immunofluorescence Analysis

Mice mammary gland cells were cultured from mammary tissues. First, the tissues were washed twice with phosphate buffered saline (PBS) and cut into 0.5 cm sections in DMEM. Next, the sediments were collected by centrifugation 1500× *g* and washed several times for 5 min each time with PBS. An aliquot of resuspended cells was manually dissociated using a 1 mL syringe with a 23 G needle and counted on a hemocytometer. Mammary gland cells were plated in DMEM containing 10% fetal bovine serum (FBS) at a density of 3.5 × 10^7^ cells/flask in a T-25 tissue culture flask for 16 h to attach to the plates. After mammary gland cell attachment, the medium was replaced with fresh DMEM containing 10% FBS, and cells were cultured for 24 or 48 h at 37 °C. Rapamycin (100 nM) was added to each well and incubated for 24 h at 37 °C. Immunodetection of hPC was then performed on mammary gland cells mounted on silanized slides. Dehydration permeabilization was performed by freezing at −20 °C in 5 mm 0.1% Triton X-100 in PBS. After blocking with 3% bovine serum albumin (BSA) in PBS, slides were incubated with a polyclonal antibody that specifically recognizes the active form of hPC in a 1:100 dilution. After washing, slides were incubated with an anti-rabbit IgG conjugated to Alexa-488. Nuclei were counter stained with 1 g/mL Hoechst 33258, and slides were mounted using fluorescent mounting medium (Dako, Carpinteria, CA, USA). Images were acquired from an Olympus AX70 fluorescence microscope.

### 4.11. ELISA Analysis of hPC

Expression levels of rec-hPC on days 1, 7, and 15 of lactation were determined by ELISA kit (Wuhan Sci. Co., Ltd., Wuhan, China). The recombinant hPC was detected using horseradish peroxidase conjugated to polyclonal antibody following 1-h incubation at 37 °C. All samples were measured in duplicates. The mean was calculated for data analysis. The level of protein was determined according to a standard curve, which takes into account four parameters. The standard curve was calculated from 0–50 ng/mL.

## 5. Conclusions

In summary, we identified Tg mice expressing the hPC gene in mammary glands. Our study demonstrated that both hPC mRNA and protein were expressed in Tg mice in the mammary glands and milk during lactation. Rec-hPC was localized in the alveolar epithelium cells of mammary glands during lactation. Our results also did not find any defective phenotypes. Thus, these Tg mouse models may allow for the production of other medicinally relevant proteins, in addition to hPC, that will confer further advantages to the medical community. Further studies will be needed to understand better the normal mammary gland development and physiology to maximize the usefulness of the mammary gland in the commercial production of recombinant proteins.
